# Comparison of Hemopoietic Biochemical Parameters in the First, Second, and Third Trimester of Pregnant Females Attending a Tertiary Care Hospital of Western Rajasthan

**DOI:** 10.7759/cureus.57766

**Published:** 2024-04-07

**Authors:** Madhu Shekhar Bissa, Jairam Rawtani, Sapna Sihag, Richa Bissa

**Affiliations:** 1 Department of Biochemistry, Dr. Bhimrao Ramji Ambedkar Government Medical College, Sirohi, IND; 2 Department of Biochemistry, Dr. Sampurnanand Medical College, Jodhpur, IND; 3 District Hospital Pratap Nagar, Dr. Sampurnanand Medical College, Jodhpur, IND

**Keywords:** hematological parameters, pregnancy, folic acid, vitamin b12, iron metabolism, maternal healthcare, biochemical parameters

## Abstract

Background: Pregnant women constitute a high-risk group for nutrient deficiency anemia which may be associated with detrimental effects on maternal and infant health.

Objectives: This study aimed to assess and compare hematological and biochemical changes across trimesters in pregnant women, considering parameters such as hemoglobin, serum iron, unsaturated iron-binding capacity (UIBC), total iron-binding capacity (TIBC), ferritin, vitamin B12, and folic acid. The research sought to identify mean value differences, correlations, and potential implications for maternal healthcare practices.

Methods: A hospital-based prospective observational study was conducted, involving 60 primigravida women with singleton pregnancies. The subjects were assessed during the first, second, and third trimesters. Biochemical parameters were assessed using standard methods, and statistical analysis was performed to identify significance and correlations.

Results: The study revealed a significant decline in hemoglobin, serum iron, ferritin, vitamin B12, and folic acid as pregnancy advanced. Hemoglobin levels decreased from 11.40 g/dl (first trimester) to 10.43 g/dl (third trimester). Serum iron exhibited a decline from 109.73 µg/dl (first trimester) to 94.03 µg/dl (third trimester). Serum ferritin decreased from 24.93 ng/ml (first trimester) to 18.21 ng/ml (third trimester). Vitamin B12 levels dropped from 255.92 pg/ml (first trimester) to 92.13 pg/ml (third trimester). Folic acid levels decreased from 13.82 ng/ml (first trimester) to 11.77 ng/ml (third trimester). UIBC and TIBC concentrations increased progressively across trimesters. Statistical evaluations confirmed the significance of these trends. The coefficient of correlation indicated positive relationships between hemoglobin and serum iron, ferritin, folic acid, and vitamin B12. Positive correlation between serum iron and ferritin, vitamin B12, and negative with folic acid. Serum ferritin negatively correlated with vitamin B12 and folic acid. Serum folic acid and vitamin B12 are positively correlated.

Conclusion: The findings emphasize the dynamic nature of hematological and biochemical changes during pregnancy. The observed trends have profound implications for maternal healthcare practices, urging targeted interventions, early monitoring, and supportive supplementation. Recognizing these variations contributes to the optimization of health outcomes for both mother and child.

## Introduction

Pregnancy in the Indian context represents a sacred and celebrated journey with cultural significance, emphasizing the well-being of both the mother and the unborn child. Recognizing the importance of comprehending hematological and endocrine variations during different trimesters is crucial for ensuring the health of pregnant women in the diverse Indian population [1,2].

The intricate balance of hematological parameters assumes heightened importance in the Indian context, where diverse dietary habits and genetic factors may influence these parameters uniquely [3]. Hemoglobin, a critical component for maternal and fetal health, undergoes necessary adaptations during pregnancy to meet increased oxygen requirements [4]. Serum iron, unsaturated iron-binding capacity (UIBC), total iron-binding capacity (TIBC), ferritin, vitamin folic acid, and cyanocobalamin together contribute to the intricate network of hematopoietic processes essential for the well-being of both the mother and the unborn child [5,6].

The rationale driving this study lies in recognizing the rich tapestry of cultural and dietary practices within the Indian population that can significantly impact hematological parameters during pregnancy. Investigating the hematological profile in Indian pregnant women across trimesters will provide valuable insights into the specific adaptations required to ensure optimal health outcomes within this diverse population [7].

This research holds profound implications for maternal healthcare practices. By elucidating the nuanced changes in hematological parameters during pregnancy, healthcare providers can tailor interventions to the unique needs of Indian pregnant women, informing targeted nutritional strategies and early interventions. The findings may contribute to reducing maternal complications and promoting the health and well-being of both mother and child.

The study's objectives were twofold: firstly, to determine differences in mean values of key hematological parameters (hemoglobin, serum iron, UIBC, TIBC, ferritin, vitamin folic acid, and cyanocobalamin) across the first, second, and third trimesters among Indian pregnant women. Secondly, the study aimed to explore inter-parameter correlations, investigating the intricate relationships between different hematological parameters, which may offer insights into early anomaly detection and facilitate proactive healthcare management.

## Materials and methods

Study setting

The present study was conducted in the Department of Biochemistry, in collaboration with the Department of Obstetrics and Gynaecology, Dr. Sampurnanand Medical College, and an associated group of Hospitals, Jodhpur. All the investigation work was done in the Clinical Biochemistry Laboratory.

Design

The study design is a hospital-based prospective observational study.

Period

The study was started after approval of the Institutional Ethical Committee [Ref No: SNMC/IEC/2022/Plan/369] and the study was carried out for 10 months from January 2023 to October 2023.

Source population

The source population was primigravida women with a singleton pregnancy, enrolled since early gestation and followed till the third trimester who visited the Antenatal Care Clinic of the Department of Obstetrics and Gynaecology Dr. Sampurnanand Medical College and its associated group of the Hospitals, Jodhpur, India, during the study period.

Inclusion criteria

Eligible participants were healthy pregnant women aged between 18 and 30 years, presenting with a singleton pregnancy. They were actively registered and attending the antenatal care (ANC) clinic, emphasizing the importance of capturing data from individuals within a specific age range and pregnancy status to maintain consistency.

Exclusion criteria

Conversely, certain criteria were established to exclude participants with potential confounding factors that could impact the study's outcomes. Pregnancies associated with pre-existing cardiac conditions such as rheumatic heart disease (RHD) or valvular heart disease, hypertension, and endocrine disorders like thyroid disorders or diabetes mellitus were not considered.

Sample size

The sample size was calculated to be a minimum of 60 primigravida females. Considering a 20-30% attrition, it was increased 80 subjects.

Blood sampling

Fasting blood samples were collected from the antecubital vein in anticoagulant and plain tubes. The serum was separated from clotted blood.

Biochemical parameters

Hemoglobin was measured using the cyanide-free sodium lauryl sulfate method (SLS), using a fully automated hematology five-part cell differential analyzer (Erba Elite 580) [8]. Serum iron and UIBC were estimated, using a fully automatic chemistry analyzer sys 1000 (Diasys) estimation of serum TIBC was calculated as the sum of serum iron and UIBC concentrations. Estimation of B12 and folic acid levels in serum was performed using chemiluminescence immunoassay (CLIA) Analyser Vitros-3600 (Ortho Clinical Diagnostics).

Statistical analysis

Statistical analyses were performed using IBM SPSS Statistics (version 23.0; IBM Corp., Armonk, NY). The arithmetic mean and the standard deviations were calculated for all the parameters studied. Student’s 't' test values and on its basis, “p” value (probability) were determined to make out the statistical significance of variance between the mean values of individual parameters among the different groups of the subject studied. The coefficient of correlation (r) between various parameters was calculated by the Pearson coefficient of correlation formula to find out the correlation of these parameters in pregnant subjects during different trimesters.

## Results

Table 1 presents the mean hemoglobin and iron concentrations of pregnant women across three trimesters. Hemoglobin levels exhibit a decreasing trend from the first (11.40 g/dl) to the third trimester (10.43 g/dl), mirroring expected physiological changes during pregnancy. Similarly, mean iron concentrations follow a decreasing pattern, with the highest in the first trimester (109.73 µg/dl) and decreasing in the second (95.46 µg/dl) and third trimesters (94.03 µg/dl). The standard deviations indicate some variability around these mean values. The mean UIBC levels exhibit a consistent increase from the first (298.54 µg/dl) to the third trimester (490.8 µg/dl), indicating an augmentation in UIBC throughout pregnancy. Similarly, the mean TIBC levels follow a rising trend, starting from the lowest in the first trimester (408.27 µg/dl) to the highest in the third trimester (584.83 µg/dl). Standard deviations suggest some variability around the mean values. Mean Serum ferritin levels exhibit a declining trend, with the highest mean in the first trimester (24.93 ng/ml) and subsequent decreases in the second (20.88 ng/ml) and third trimesters (18.21 ng/ml). A similar pattern was observed for vitamin B12 and folic acid, with the highest means in the first trimester (vitamin B12: 255.92 pg/ml, folic acid: 13.82 ng/ml) followed by reductions in the second (vitamin B12: 102.37 pg/ml, folic acid: 11.47 ng/ml) and third trimesters (vitamin B12: 92.13 pg/ml, folic acid: 11.77 ng/ml). Standard deviations indicate variability in these micronutrient levels. A gradual decline in mean hemoglobin levels is evident from the first (11.40 g/dl) to the third trimester (10.43 g/dl), paralleled by a reduction in serum iron levels from 109.73 µg/dl to 94.03 µg/dl. Concurrently, UIBC and TIBC exhibit an increasing trend, rising from 298.54 µg/dl to 490.8 µg/dl and from 408.27 µg/dl to 584.83 µg/dl, respectively. Mean serum ferritin, vitamin B12, and folic acid levels consistently decrease across trimesters, indicating a decline in iron stores and essential vitamins during pregnancy.

**Table 1:** Comparison of hemopoietic biochemical parameters in pregnant female subjects during different trimesters of pregnancy g/dl: grams per deciliter; µg/dl: microgram per deciliter; ng/ml: nanograms per milliliter; pg/ml: picogram per milliliter

Parameters Studied	Trimester of Pregnancy (Mean + SD)		
	FIRST	SECOND	THIRD
Hemoglobin (g/dl)	11.40 ± 1.04	10.63 ± 0.99	10.43 ± 0.90
Serum Iron (µg/dl)	109.73 ± 21.68	95.46 ± 18.86	94.03 ± 18.58
Serum UIBC (µg/dl)	298.54 ± 56.26	358.25 ± 67.51	490.8 ± 92.49
Serum TIBC (µg/dl)	408.27 ± 62.08	408.27 ± 62.08	584.83 ± 95.96
Serum Ferritin (ng/ml)	24.93 ± 4.73	20.88 ± 3.99	18.21 ± 3.34
Serum Vitamin B12(pg/ml)	255.92 ± 46.94	102.37 ± 18.78	92.13 ± 16.90
Serum Folic Acid (ng/ml)	13.82 ± 2.50	11.47 ± 2.10	11.77 ± 2.13

Table 2 presents the statistical analysis of hemoglobin, serum iron, UIBC, and TIBC concentrations among different trimesters of pregnancy. Significantly higher values are observed in the first trimester compared to the second and third trimesters for hemoglobin and iron (p<0.001), indicating a decrease as pregnancy progresses. Statistically highly significant differences are observed in UIBC and TIBC across all trimester comparisons (p<0.001), highlighting dynamic changes in iron-binding capacities. However, no significant differences were observed between the second and third trimesters for hemoglobin and serum iron (p>0.05), suggesting stability in these parameters during these stages. Highly significant differences are observed in serum ferritin levels between the first and second trimesters (t-value: 5.07, p<0.001) and the first and third trimesters (t-value: 8.99, p<0.001), indicating substantial changes in iron stores. Vitamin B12 levels show highly significant differences between all trimester pairs (p<0.001), highlighting variations in this essential vitamin throughout pregnancy. Folic acid, on the other hand, exhibits significant differences only between the first and second trimesters (t-value: 5.58, p<0.001).

**Table 2:** Statistical analysis of blood hemoglobin, serum iron, UIBC, TIBC, ferritin, vitamin B12, and folic acid concentrations among different trimesters of pregnancy p≤0.05 is considered as statistically significant; NS: non-significant; HS: highly significant; VS: very significant; UIBC: unsaturated iron-binding capacity; TIBC: total iron-binding capacity

	Hemoglobin	Iron	Serum UIBC	Serum TIBC	Serum Ferritin	Vitamin B12	Folic Acid
Trimester Compared	p-value	p-value	p-value	p-value	p-value	p-value	p-value
I v/s II	p<0.001 (HS)	p<0.001 (HS)	p<0.001 (HS)	p<0.001 (HS)	p<0.001 (HS)	p<0.001 (HS)	p<0.001 (HS)
I v/s III	p<0.001 (HS)	p<0.001 (HS)	p<0.001 (HS)	p<0.001 (HS)	p<0.001 (HS)	p<0.001 (HS)	p<0.001 (HS)
II v/s III	p>0.05 (NS)	p>0.05 (NS)	p<0.001 (HS)	p<0.001 (HS)	p<0.001 (HS)	p<0.01 (VS)	p>0.05 (NS)

Table 3 displays coefficients of correlation (r) among studied hematological and biochemical parameters across trimesters of pregnancy. Positive correlations are observed, between hemoglobin vs. iron (r ranging from 0.018 to 0.057), hemoglobin vs. ferritin (r ranging from 0.141 to 0.213), and iron vs. vitamin B12 (r ranging from 0.046 to 0.057), indicating concurrent changes in these parameters throughout gestation. Conversely, negative correlations were seen in iron vs. folic acid (r ranging from -0.205 to -0.310) and ferritin vs. vitamin B12 (r ranging from -0.210 to -0.311), suggesting indirect relationships, indicating that changes in one parameter coincide with decreases in the other.

**Table 3:** Comparison of Pearson’s coefficient of correlation (r) between various parameters studied p≤0.05 is considered as statistically significant

Parameter Compared	Coefficient of Correlation (R) Values			Correlation
	First trimester	Second trimester	Third trimester	
Hemoglobin v/s iron	0.018	0.057	0.026	Positive
Hemoglobin v/s ferritin	0.141	0.185	0.213	Positive
Hemoglobin v/s folic acid	0.181	0.171	0.294	Positive
Hemoglobin v/s vitamin B12	0.077	0.081	0.027	Positive
Iron v/s ferritin	0.231	0.156	0.154	Positive
Iron v/s folic acid	-0.205	-0.225	-0.310	Negative
Iron v/s vitamin B12	0.046	0.053	0.057	Positive
Ferritin v/s folic acid	-0.192	-0.220	-0.210	Negative
Ferritin v/s vitamin B12	-0.210	-0.280	-0.311	Negative
Folic acid v/s vitamin B12	0.138	0.139	0.135	Positive

## Discussion

The study systematically examined hematological and biochemical parameters across different trimesters of pregnancy shedding light on the nuanced changes that occur during this critical period as deficiency occurs due to deterioration of nutritional balance during pregnancy.

In the present study, a notable decline in hemoglobin levels was observed with the progression of pregnancy trimesters. Similarly, the levels of serum iron and serum ferritin also exhibited a decreasing trend as pregnancy advanced, and this difference reached statistical significance in comparisons between the 1st vs 2nd, 1st vs 3rd, and 2nd vs 3rd trimesters, particularly in serum ferritin. However, serum iron and hemoglobin showed non-significant changes as pregnancy advanced from the 2nd to the 3rd trimester.

Regarding mean serum UIBC and TIBC concentrations, there was a consistent increase from the 1st trimester to the 3rd trimester, and this difference was found to be statistically significant in the 1st vs 2nd, 1st vs 3rd, and 2nd vs 3rd-trimester comparisons. Consequently, our study concludes that hemoglobin, iron, and ferritin tend to decrease, while serum UIBC and TIBC tend to increase as pregnancy progresses through trimesters.

Our study aligns with the findings of Talabani, revealing a statistically significant declining trend in hemoglobin levels from the 1st to the 3rd trimester, accompanied by an increase in TIBC as pregnancy progresses [9]. This is consistent with the observations made by Tan et al. who noted a decrease in both hemoglobin and ferritin levels with advancing trimesters, particularly among women with multiple gestations [10].

Narasamma et al. further support these trends, emphasizing a statistically significant difference in hemoglobin, serum iron, ferritin, UIBC, and TIBC concentrations across the three trimesters of pregnancy [11]. Aloy-Amadi et al. concur with our results, reporting a significant decrease in serum ferritin and serum iron, and a notable increase in TIBC from the first to the third trimester [12].

Churchill et al. documented a decrease in hemoglobin levels from the 1st to the 3rd trimester [13]. However, Satué et al. presented a contrasting perspective, reporting significant increases in iron (Fe), ferritin, and TIBC concentrations, a decrease in UIBC, and no changes in hemoglobin levels as pregnancy progresses [14].

Indeed, the observed variations in iron-related parameters across trimesters could be attributed to the remarkable physiological demand for iron during pregnancy [15]. The escalating iron requirement is intricately linked to gestational age, surging from 0.8 mg/day in early pregnancy to a substantial 7.5 mg/day in late pregnancy. Consequently, an average iron requirement of 4.4 mg/day is conventionally acknowledged throughout pregnancy. This substantial increase in iron demand reflects the intricate processes associated with fetal development and maternal adaptation, underscoring the dynamic nature of iron metabolism during the various stages of gestation [16].

The observed decline in hemoglobin, iron, and ferritin levels during pregnancy may be attributed to the physiological expansion of fluid (plasma), which experiences a significant increase of approximately 50%, peaking around the 32nd week of gestation. This heightened plasma volume leads to the dilution of red blood cells, resulting in a reduction in their concentration. Consequently, the increased demand for iron during pregnancy is crucial for expanding the red blood cell mass and supporting fetal and placental growth. This augmented requirement for iron prompts the mobilization of ferritin from its stores, as documented by Narasamma et al. [11]. Moreover, a study by Evanchuk et al. [17]. reported an elevated prevalence of maternal iron deficiency throughout pregnancy, with 61% exhibiting depleted iron stores.

In our study, pregnant females exhibited a decreasing trend in the mean levels of vitamin B12 and folic acid from the 1st trimester to the 3rd trimester. The analysis revealed a statistically significant difference in the mean serum levels of vitamin B12 and folic acid when comparing the 1st vs 2nd trimester and 1st vs 3rd trimester. However, in the comparison between the 2nd and 3rd trimesters, the level of vitamin B12 showed a significant difference, unlike folic acid.

Consistent with our findings, Singh observed a decline in mean vitamin B12 and serum folate levels from the 1st to the 3rd trimester, with a statistically significant difference [18]. Beyoglu and Kostu reported a significant difference in vitamin B12 levels during the 1st trimester compared to the 2nd and 3rd trimesters, while the 2nd and 3rd trimesters did not exhibit a significant difference [19]. Lai et al. reported a 56% rate of vitamin B12 insufficiency or deficiency in their study [20], while Du et al. found vitamin B12 deficiency in 69.6% of pregnant women [21].

Similarly, Meena et al. reported a steady decline in maternal plasma folate concentration and vitamin B12 concentration [22]. According to Beyoglu and Kostu, B12 deficiency during pregnancy may be attributed to the utilization of vitamin B12 and folic acid in DNA synthesis for organogenesis in infant development [19]. The low levels of folic acid and B12 in pregnant women may be influenced by socioeconomic status and physiological changes during pregnancy. Developing countries often experience high rates of micronutrient nutritional deficits among pregnant women [23]. Physiologically, the reduction in vitamin B12 levels during pregnancy can be attributed to hormonal changes, increased blood hemodilution, active vitamin transport to the fetus, decreased albumin, increased glomerular filtration, and alterations in vitamin B12-binding protein capacity and saturation [24].

Clinical implications

Understanding the hematological changes in Indian pregnant women is crucial for tailoring healthcare interventions to the specific needs of this diverse population. The study's implications for maternal healthcare practices in India are profound. The observed variations emphasize the necessity of targeted nutritional strategies and early interventions to mitigate complications arising from deficiencies in iron, vitamin B12, and folic acid.

Limitations

The study included women with a singleton pregnancy. Including multipara women in research studies on maternal health parameters during pregnancy can indeed provide valuable insights into the potential long-term effects of pregnancy on female health. Previous pregnancies can have a significant impact on various aspects of maternal health, including hematological parameters, metabolic health, and cardiovascular function. By considering the experiences of multipara women, we can develop more comprehensive approaches to promoting maternal health and well-being across the reproductive lifespan. The number of pregnant women in each trimester group may be small, leading to limited statistical power. The study results cannot be generalized to the general pregnant population since the study was conducted on a hospital-based population.

## Conclusions

In the Indian scenario, there is a high prevalence of nutrient deficiencies during pregnancy due to multiple factors such as high parity, poor dietary habits, low socioeconomic, status less awareness, etc. In conclusion, hematological and biochemical changes during pregnancy offer a proper foundation and should be monitored and cared for regularly replenishment of essential nutrients. Further research and intervention ultimately aiming to enhance maternal and fetal health outcomes in the diverse landscape of pregnancy should be carried out.
